# New Appliances and Things Medical

**Published:** 1901-11-23

**Authors:** 


### NEW APPLIANCES AND THINGS MEDICAL.

**[We shall be glad to receive, at our Office, 28 & 29 Southampton Street, Strand, London, W.C., from the manufacturers, specimens of all new preparations and appliances which may be brought out from time to time.]**

### HARTMANN'S WOOD WOOL VACCINATION PADS.

(The Sanitary Wood Wool Company, Limited, 26 Thavies Inn, London, E.C.)

It is always a matter of difficulty to fix a satisfactory dressing to a vaccinated arm, whether in the case of a child or an adult individual. At the present day public vaccinators are compelled by law, and private practitioners by public opinion, to apply a dressing not only after the formation of vesicles but at the time of the inoculation also. Many simple devices have been contrived to protect the arm from accidental contamination, but none with which we are acquainted meet the necessities of the case more completely than the pad of wood wool which is manufactured by the Sanitary Wood Wool Company. The pads are formed of woodwool contained between layers of tissue with an opening cut in the latter where it comes in contact with the seat of the inoculation. The advantage of this is that there can be no tearing off of the seat in the process of removing or changing the dressing. A small particle of wool may, perhaps, adhere to the surface of the wound, but offers no resistance to the removal of the pad. The tapes are so arranged that two are tied round the arm, and one passes over the shoulder and below the arm of the opposite side. The price is only 3d. per pad, and the usefulness of this simple dressing is beyond dispute.

### MAIGNEN'S PATENT "FILTRE RAPIDE."

(Maignen's Filtre Rapide and "Anti-Calcaire" Company, 15 Great Marlborough Street, London, W.)

This well-known filter possesses certain advantages which ought to weigh with those who wish to ensure a supply of palatable and well-clarified drinking water. The most important of its advantages seems to us to be the facility with which it can be taken to pieces and each part boiled so as to destroy those fungi or algæ which are so apt to grow in filters which cannot be so treated. The makers state that this filter arrests the germs of disease, and this is probably true for a time. At the present day, however, but little, perhaps no, faith is put in the domestic filtration of water as a means of arresting disease germs, and the makers of this filter very properly recommend that water for drinking should be boiled before filtration.

